# Immunomodulatory Activity of Octenyl Succinic Anhydride Modified Porang (*Amorphophallus oncophyllus*) Glucomannan on Mouse Macrophage-Like J774.1 Cells and Mouse Primary Peritoneal Macrophages

**DOI:** 10.3390/molecules22071187

**Published:** 2017-07-15

**Authors:** Sellen Gurusmatika, Kosuke Nishi, Eni Harmayani, Yudi Pranoto, Takuya Sugahara

**Affiliations:** 1 Department of Bioscience, Graduate School of Agriculture, Ehime University, Matsuyama, Ehime 790-8566, Japan; sgurusmatika@gmail.com (S.G.); nishi.kosuke.mx@ehime-u.ac.jp (K.N.); 2 Faculty of Agricultural Technology, Gadjah Mada University, Bulaksumur, Yogyakarta 55281, Indonesia; eniharmayani@yahoo.com (E.H.); pranoto@ugm.ac.id (Y.P.); 3 Food and Health Sciences Research Center, Ehime University, Matsuyama, Ehime 790-8566, Japan; 4 South Ehime Fisheries Research Center, Ehime University, Ainan, Ehime 798-4292, Japan

**Keywords:** porang glucomannan, octenyl succinic anhydride, macrophage, cytokine, phagocytosis activity, J774.1 cell

## Abstract

Porang is a local plant of Indonesia, which has a high content of glucomannan. In this study, porang glucomannan (PG) was esterified with octenyl succinic anhydride (OSA) to enhance emulsion properties to be widely used in food industry. OSA-modified PG (OSA-PG) enhanced the phagocytosis activity of macrophage-like J774.1 cells and mouse peritoneal macrophages. In addition, OSA-PG increased the production of IL-6 and TNF-α by enhancing their gene expression. Immunoblot analysis displayed that OSA-PG tended to activate both nuclear factor-κB and mitogen-activated protein kinase cascades. Treatment of OSA-PG with polymyxin B revealed that cytokine production induced by OSA-PG was not caused by endotoxin contamination. Our findings also indicated that OSA-PG activates macrophages through not only Toll-like receptor (TLR) 4, but another receptor. Overall findings suggested that OSA-PG has a potential as an immunomodulatory food factor by stimulating macrophages.

## 1. Introduction

Porang (*Amorphophallus oncophyllus*) tuber is a potential glucomannan source in Indonesia due to its high-level glucomannan content. The tuber is cultivated in Indonesia as a secondary crop under the teak, mahogany, and sonokeling plantation. As a source of glucomannan, porang may offer many benefits as a functional food ingredient [1,2]. Glucomannans are comprised of mannose and glucose with some acetylated residues. The ratio of mannose to glucose, the acetylation degree, and the molecular weight could be different depending on the origin of the glucomannan source [3,4].

Glucomannan has been widely used in food industry as thickening, gelling, texturing, water-binding, and emulsion-stabilizing agents [5,6,7]. Emulsion is an important structure in a number of daily products such as manufactured foods, pharmacy, cosmetics, and agrochemicals. Esterification of glucomannan with octenyl succinic anhydride (OSA) produces a kind of polymeric surfactant with a high potential to be used as an emulsion stabilizer in foods. Recently, OSA modification was introduced into konjac glucomannan, yielding a hydrophilic surfactant with a favorable surface activity compared with native konjac glucomannan and OSA-modified starch [8]. OSA can also react with other natural polysaccharides such as starch, gum arabic, pectin, and β-cyclodextrin in order to improve emulsifying capacity [9,10,11,12].

Evidence of the beneficial functionality of konjac glucomannan has been obtained in recent years [13], which supports the role as health-promoting foods such as a weight loss food by filling the stomach and bringing a sense of satiety [14]. In addition, konjac glucomannan has been reported to alleviate hyperglycemia and hypercholesterolemia [15], to selectively promote the growth of probiotic bacteria [16], and to contribute to immunoregulation [17,18]. However, there is no paper reporting the effect of porang glucomannan (PG) on the immune system.

The immune response can be divided into two categories: the innate immune response and the adaptive immune response. Macrophages are a member of the innate immune system and are versatile cells that play many roles. A major function of macrophages is phagocytosis, a process to present fragmented pathogens as an antigen on their cell surface and to initiate activation of the adaptive immune response [19,20]. Activated macrophages are characterized by the increase in phagocytosis activity and secretion of cytokines such as tumor necrosis factor (TNF) α and interleukin (IL) 6 to recruit and activate other leukocytes, resulting in the activation of the adaptive immune response and the establishment of protective immunity [21].

In this study, we investigated the immunomodulatory effect of OSA-modified PG (OSA-PG) on mouse macrophage-like J774.1 cells and mouse primary peritoneal macrophages. The effects of OSA-PG on phagocytotic activity, proinflammatory cytokine production, and the activation of mitogen-activated protein kinases (MAPK) are described in this study. The findings shown in this paper may generate further value in expanding porang tuber use in the food industry as a functional food.

## 2. Results and Discussion

### 2.1. Effect of OSA-PG on Cytokine Production by J774.1 Cells and Peritoneal Macrophages

**Figure 1:**
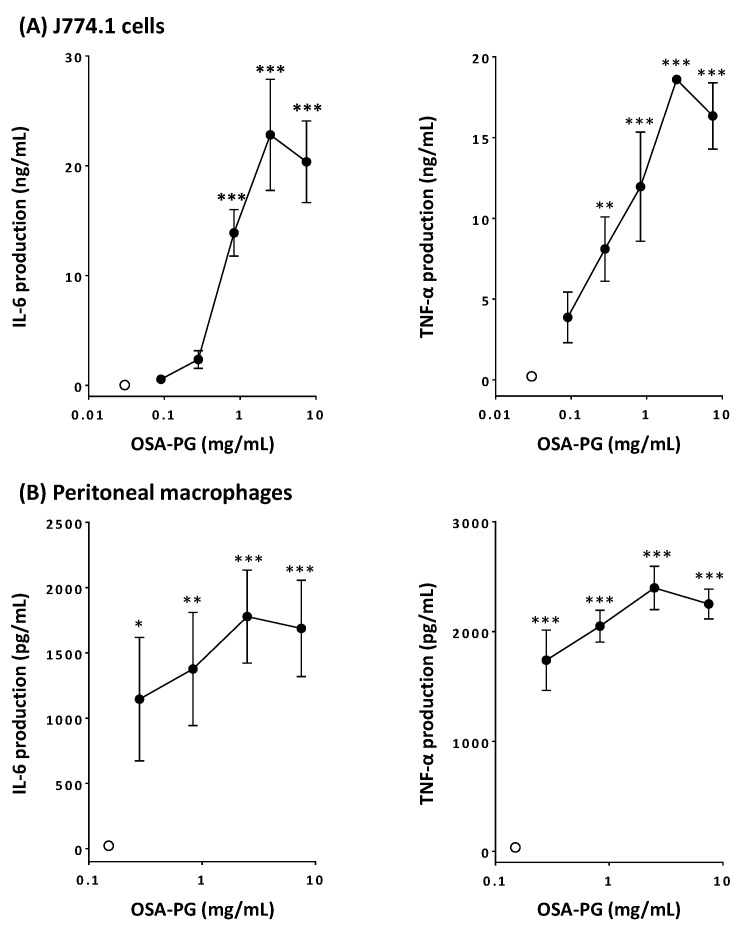
Effect of octenyl succinic anhydride-modified porang glucomannan (OSA-PG) on cytokine production by J774.1 cells (**A**) and peritoneal macrophages (**B**). Cells were treated with various concentrations of OSA-PG for 6 h, and the concentrations of tumor necrosis factor (TNF) α and interleukin (IL) 6 in the culture medium were measured. Open circles represent the cells untreated with OSA-PG (control). Data were represented as mean ± standard deviation of three independent experiments. * *p* < 0.05, ** *p* < 0.01, *** *p* < 0.001 against control by Dunnett’s multiple comparison test.

Activation of macrophages leads to increase in the phagocytosis activity and enhanced production of cytokines such as TNF-α, interferon (IFN) γ, IL-1, IL-6, IL-10, IL-12, and so on [22,23]. Thus, the effect of OSA-PG on macrophage-like J774.1 cells and peritoneal macrophages was analyzed by evaluating the production of TNF-α and IL-6. Production of both IL-6 and TNF-α significantly increased in concentration-dependent manners by treating J774.1 cells with OSA-PG, whereas untreated J774.1 cells secreted very little amounts of cytokines (Figure 1A). When J774.1 cells were treated with OSA-PG at the concentration of 2.5 mg/mL, the production of IL-6 and TNF-α increased approximately 600- and 80-fold, respectively, compared with control. Cytokine production by J774.1 cells decreased when the cells were treated at the highest concentration, although no cytotoxicity of OSA-PG was observed (supplemental material). Thus, the concentration (7.5 mg/mL) seemed to exert an adverse effect on J774.1 cells. We also investigated the effect of OSA-PG on peritoneal macrophages in the same way. As a result, OSA-PG showed similar effects on peritoneal macrophages. As displayed in Figure 1B, IL-6 and TNF-α production significantly increased 70- and 60-fold, respectively, compared with control when peritoneal macrophages were treated with OSA-PG at the concentration of 2.5 mg/mL. As is the case with J774.1 cells, cytokine production by peritoneal macrophages declined when the cells were treated at the highest concentration of OSA-PG (7.5 mg/mL). These findings suggested that OSA-PG exhibits a remarkable immunomodulatory activity by upregulating the secretion of IL-6 and TNF-α not only by a macrophage-like cell line, but also by primary macrophages. As proinflammatory mediators, IL-6 and TNF-α play an important role in maintaining the appropriate and efficient activity of the immune system in response to surrounding immunological challenges [24]. TNF-α is a cytokine secreted mainly by macrophages. IL-6 is considered as a major immune and inflammatory mediator [25]. For further experiments, OSA-PG was used at the concentration of 2.5 mg/mL, at which the highest effect was observed in the cytokine production assay. Because OSA-PG is not digested in the human digestive tract, we do not need to think about the degradation of OSA-PG. Depending on the amount of OSA-PG in foods, we assume that such a concentration of OSA-PG can be achieved in our intestine.

### 2.2. Effect of OSA-PG on Transcription of Cytokine Genes in J774.1 Cells and Peritoneal Macrophages

**Figure 2:**
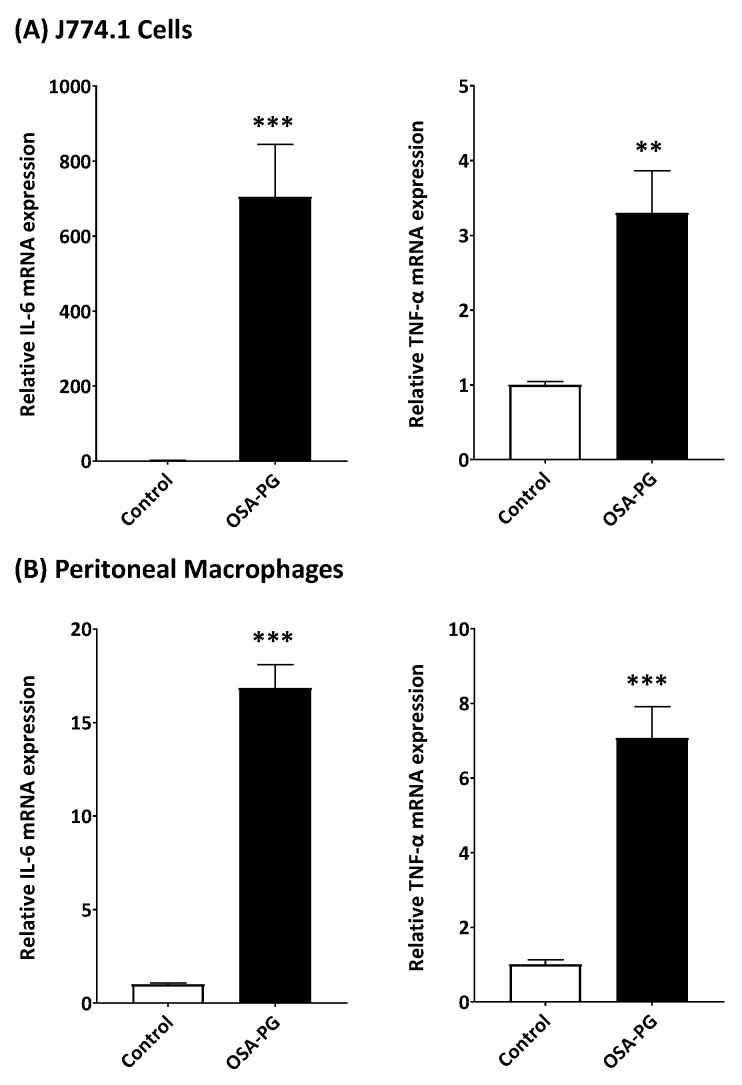
Effect of octenyl succinic anhydride-modified porang glucomannan (OSA-PG) on transcription levels of cytokine genes in J774.1 cells (**A**) and peritoneal macrophages (**B**). Cells were treated with/without OSA-PG (2.5 mg/mL) for 6 h, and the mRNA levels of tumor necrosis factor (TNF) α and interleukin (IL) 6 were evaluated by real-time RT-PCR. Data were represented as mean ± standard deviation of three independent experiments. ** *p* < 0.01, *** *p* < 0.001 against control by unpaired *t*-test.

Although OSA-PG showed the significantly enhanced cytokine production by J774.1 cells and peritoneal macrophages, it was necessary to understand how the cytokine production was stimulated by OSA-PG. We thus evaluated the gene expression levels of IL-6 and TNF-α. As shown in Figure 2A, OSA-PG significantly induced the transcription of IL-6 and TNF-α genes compared with the control in J774.1 cells. The mRNA levels of IL-6 and TNF-α genes increased 620- and 4-fold, respectively, compared with control by treating J774.1 cells with 2.5 mg/mL of OSA-PG. As with the result using J774.1 cells, the transcription of IL-6 and TNF-α genes was also elevated in peritoneal macrophages by treating with OSA-PG as shown in Figure 2B. The mRNA levels of IL-6 and TNF-α increased 15- and 4-fold, respectively, compared with control by treating peritoneal macrophages with 2.5 mg/mL of OSA-PG. These results indicated that OSA-PG stimulates the cytokine production by enhancing cytokine gene expression. These findings further support the notion that OSA-PG is a potent macrophage activator.

### 2.3. Effect of OSA-PG on Phagocytosis Activity of J774.1 Cells

**Figure 3:**
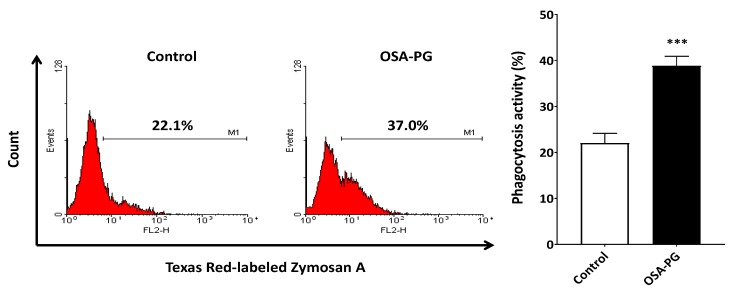
Effect of octenyl succinic anhydride-modified porang glucomannan (OSA-PG) on phagocytosis activity of J774.1 cells. J774.1 cells were treated with/without OSA-PG (2.5 mg/mL) for 6 h, and incubated with Texas Red-labeled zymosan A for 1 h. Phagocytosis activity was then measured in a flow cytometer. A representative histogram from three independent experiments is shown. A graph represents mean ± standard deviation of three independent experiments. *** *p* < 0.001 against control by unpaired *t*-test.

Phagocytosis is of vital importance in the immune system. Thus, the effect of OSA-PG on phagocytosis activity of J774.1 cells was evaluated to study the immunomodulatory activity of OSA-PG. J774.1 cells were treated with OSA-PG, and the Texas Red-labeled zymosan A was then added to the culture medium. As a result, J774.1 cells treated with OSA-PG showed higher phagocytosis activity against zymosan A than the control as depicted in Figure 3. This finding indicated that OSA-PG possesses an immunomodulatory effect to activate phagocytosis activity of J774.1 cells. Phagocytosis by macrophages is critical in the process to remove and degrade pathogenic materials in the body. It requires recognition through a variety of germ-line encoded pattern recognition receptors (PRRs), including Toll-like receptors (TLRs), that bind to conserved molecular patterns on microbes, referred to as pathogen-associated molecular patterns. Moreover, stimulation of PRRs initiates intracellular signaling pathways and eventually induces the expression of cytokine genes [21,26,27].

### 2.4. Effect of OSA-PG on Nuclear Factor-κB (NF-κB) and MAPK Signaling Cascades

**Figure 4:**
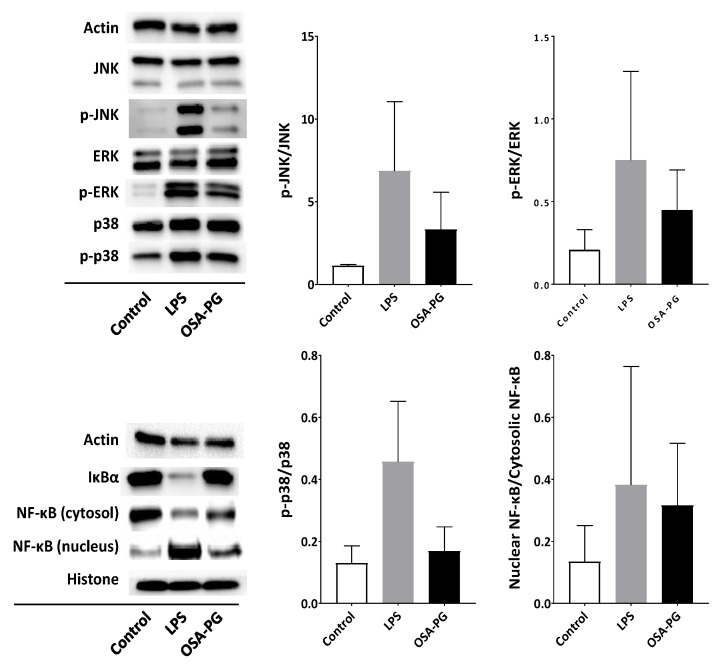
Effect of octenyl succinic anhydride-modified porang glucomannan (OSA-PG) on nuclear factor-κB (NF-κB) and mitogen-activated protein kinase signaling cascades. J774.1 cells were treated with OSA-PG (2.5 mg/mL), lipopolysaccharides (LPS; 200 ng/mL), or neither of them (control) for 30 min, and the protein levels were evaluated by immunoblot analysis. The p-ERK, p-JNK, and p-p38 represent phosphorylated ERK, phosphorylated JNK, and phosphorylated p38, respectively. A representative blot from three independent experiments is shown. The result of densitometric analysis is expressed as the ratio of (phosphorylated protein amount)/(whole protein amount) or (nuclear protein amount)/(cytosolic protein amount). Data are expressed as mean ± standard deviation of three independent experiments. No statistical significance was found by Dunnett’s multiple comparison test.

TLR4 is involved in the activation of MAPK and NF-κB signal transduction pathways. Activation of MAPK and NF-κB signaling cascades eventually leads to the expression of proinflammatory cytokine genes [28,29]. Therefore, the effect of OSA-PG on NF-κB and MAPK signaling cascades was evaluated by immunoblot analysis to understand the mechanism of macrophage activation by OSA-PG. As displayed in Figure 4, OSA-PG tended to facilitate NF-κB translocation into the nucleus. OSA-PG also tended to activate MAPK signaling cascades by enhancing phosphorylation of c-Jun N-terminal kinase (JNK), extracellular signal-regulated protein kinases (ERK), and p38 MAPK in J774.1 cells. These results revealed that OSA-PG may elevate the gene expression levels of cytokines via activating NF-κB and MAPK signaling cascades.

Polysaccharides have been suggested to be capable of enhancing the immune system by stimulating natural killer (NK) cells, T cells, B cells, and macrophages-dependent immune responses [30]. It is thought that plant polysaccharides may stimulate macrophages and other innate immune cells by binding to one or more cell surface receptors, including TLR2, TLR4, CD14, CR3, scavenger receptor, dectin-1, and mannose receptors [22]. Activation of TLR4 leads to the subsequent signal transduction such as NF-κB and MAPK signalings. NF-κB regulates a number of transcription factors involved in the proinflammatory responses. In resting cells, NF-κB is bound to IκBα, an inhibitor of NF-κB, and remains inactive in the cytoplasm. Activation of NF-κB leads to the phosphorylation of IκBα, releasing NF-κB in the active state. The active NF-κB subsequently translocates into the nucleus and initiates transcription of its target genes. Phosphorylated IκBα is used as an indicator of the activation of NF-κB signaling cascade. Therefore, NF-κB activation is indicated by a decrease of NF-κB in cytoplasm, an increase in phosphorylated IκBα in cytoplasm, and an increase in NF-κB in the nucleus due to its translocation from cytoplasm [28]. Figure 4 shows that OSA-PG treatment tended to reduce NF-κB in the cytoplasm and increased NF-κB in the nucleus as indicated in bands of immunoblot analysis, implying that OSA-PG might activate the TLR4 signaling pathway.

MAPK signaling cascade, as well as NF-κB, is also responsible for the immune responses. MAPKs, mainly composed of three subfamilies—including ERK, JNK, and p38—are serine/threonine-specific protein kinases, which can be triggered by various extracellular stimuli such as mitogens and proinflammatory cytokines [31]. OSA-PG activated phosphorylation of ERK, JNK, and p38 as shown in Figure 4. The activation of JNK contributes to response to various extracellular stimuli—such as TNF, epidermal growth factor, platelet-derived growth factor, transforming growth factor β, and lysophosphatidic acid—as well as diverse environmental stresses. JNK and p38 are generally thought to play roles in inflammation, differentiation, apoptosis, and insulin resistance [32,33]. Our results shows that ERK, JNK, and p38 MAPK signaling pathways tended to be activated in OSA-PG-treated J774.1 cells. In addition, activation of NF-κB and MAPK cascades is involved in TLR-induced phagocytosis activity of macrophages.

### 2.5. Effect of Polymyxin B-Treated OSA-PG on Cytokine Production

**Figure 5:**
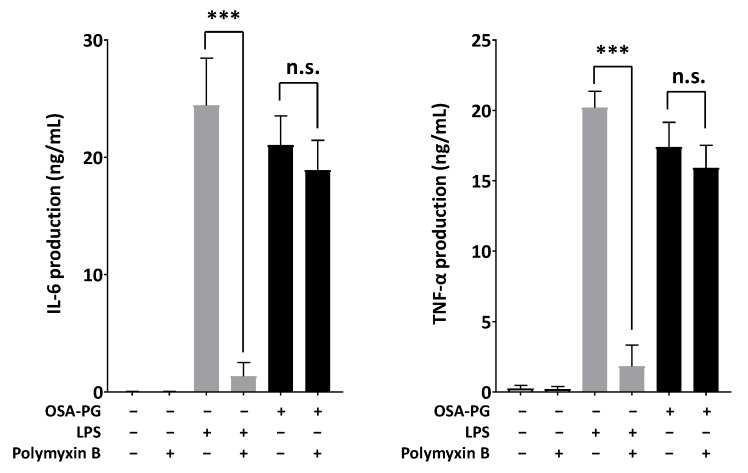
Effect of polymyxin B-treated octenyl succinic anhydride-modified porang glucomannan (OSA-PG) on cytokine production. J774.1 cells were treated with polymyxin B-treated OSA-PG (2.5 mg/mL), polymyxin B-treated lipopolysaccharides (LPS; 200 ng/mL), or neither of them for 6 h, and the concentrations of tumor necrosis factor (TNF) α and interleukin (IL) 6 in the culture medium were measured. Data were represented as mean ± standard deviation of three independent experiments. *** *p* < 0.001 against control by unpaired *t*-test. n.s. represents not statistically significant.

Polymyxin B, an antagonist of endotoxin, was used to evaluate whether endotoxin was present in OSA-PG and contributed to the immunomodulatory activity of OSA-PG. To address this possible interference, OSA-PG was mixed with polymyxin B and used for the cytokine production assay to observe whether there was significant reduction in IL-6 and TNF-α production. Polymyxin B is a polycationic antibiotic with a high affinity for the lipid A component of lipopolysaccharides (LPS), which results in neutralization of endotoxin-like bioactivity of most forms of LPS [34]. As shown in Figure 5, polymyxin B markedly inhibited both IL-6 and TNF-α production induced by LPS (reduced >90%). However, it had no effect on OSA-PG-induced production of IL-6 (reduced ~10%) and TNF-α (reduced ~8%), indicating that the proinflammatory cytokine production induced by OSA-PG was not caused by contaminated endotoxin.

### 2.6. Effect of OSA-PG on TLR4

**Figure 6:**
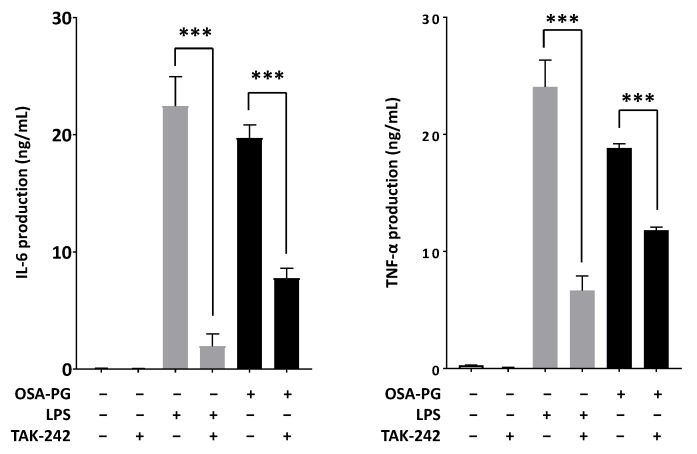
Effect of octenyl succinic anhydride-modified porang glucomannan (OSA-PG) on Toll-like receptor 4 in J774.1 cells. J774.1 cells were treated with/without 0.2 μM TAK-242 for 1 h. The cells were then treated with OSA-PG (2.5 mg/mL), lipopolysaccharides (LPS; 200 ng/mL), or neither of them for 6 h, and the concentrations of tumor necrosis factor (TNF) α and interleukin (IL) 6 in the culture medium were measured. Data were represented as mean ± standard deviation of three independent experiments. *** *p* < 0.001 against control by unpaired *t*-test.

TLRs are one of PRRs that recognize microbial pathogens and their components and initiate the activation of intracellular signaling such as NF-κB and MAPK signaling cascades, resulting in the release of a wide variety of inflammatory mediators including nitric oxide and cytokines such as IL-6, TNF-α, and IFN [29]. Thus, we examined the effect of OSA-PG on TLR4 using ethyl (6*R*)-6-[*N*-(2-chloro-4-fluorophenyl)sulfamoyl]cyclohex-1-ene-1-carboxylate (TAK-242), a small-molecule inhibitor specific for TLR4. As shown in Figure 6, treating J774.1 cells with TAK-242 inhibited IL-6 and TNF-α production induced by LPS or OSA-PG. The inhibition rates of LPS were 91% for IL-6 production and 72% for TNF-α production, whereas those of OSA-PG were around 60% for IL-6 production and 40% for TNF-α production. These data revealed that the effect of OSA-PG was not completely inhibited by TAK-242. We hypothesized that OSA-PG stimulates cytokine production by acting not only on TLR4, but also on another receptor.

OSA-PG is water-soluble and heat-stable with the molecular weight higher than 14,000 (data not shown). Glucomannan is a polysaccharide comprised of mannose and glucose sugars. In this study, PG was modified by succinylation using an OSA reagent in order to increase emulsion ability. Succinylation leads to the replacement of hydroxyl groups in the glucomannan molecules with carbonyl groups of OSA [8]. In recent years, the immunomodulatory effect of various polysaccharides and oligosaccharides from food sources showed their potential as immunotherapeutic agents [22,30,31,35]. Some botanical polysaccharides have been found to enhance the viability of macrophages against pathogenic microorganisms and tumorigenesis by increasing the secretion of nitric oxide and the production of cytokines. In addition, polysaccharides have been reported to bind to specific membrane receptors on macrophages and to activate the immune response through distinct signal transduction pathways [36,37]. For instance, chitooligosaccharides and *O*-acetyl-glucomannan have been reported to be capable of stimulating macrophage activation to reduce various inflammatory mediators, including TNF-α, IL-6, and NO [38,39].

## 3. Materials and Methods

### 3.1. Reagents

2-Octen-1-ylsuccinic anhydride, RPMI 1640 medium, fetal bovine serum (FBS), penicillin, streptomycin, polymyxin B sulfate salt, and LPS from *Escherichia coli* 026/B6 were purchased from Sigma-Aldrich (St. Louis, MO, USA). Enzyme-linked immunosorbent assay (ELISA) kits for TNF-α and IL-6 were purchased from R&D Systems (Minneapolis, MN, USA) and Biolegend (San Diego, CA, USA), respectively. Goat anti-actin antibody and anti-goat IgG antibody labeled with horseradish peroxidase (HRP) were purchased from Santa Cruz Biotechnology (Santa Cruz, CA, USA). Mouse anti-IκBα antibody, HRP-labeled anti-mouse IgG antibody, HRP-labeled anti-rabbit IgG antibody, and rabbit antibodies against histone H3, NF-κB p65, ERK1/2, phosphorylated ERK1/2, JNK, phosphorylated JNK, p38 MAPK, and phosphorylated p38 MAPK were purchased from Cell Signaling Technology (Danvers, MA, USA). TAK-242 was purchased from Chemscene (Monmouth Junction, NJ, USA).

### 3.2. Cells and Cell Culture

Mouse macrophage-like cell line J774.1 cells were obtained from the Japanese Collection of Research Bioresources Cell Bank (Osaka, Japan) and maintained in RPMI 1640 medium supplemented with 100 U/mL of penicillin, 100 μg/mL of streptomycin, and 10% FBS at 37 °C under humidified 5% CO_2_. Mouse peritoneal macrophages were prepared as described previously [40] with some modifications. Briefly, eight-week-old female BALB/c mice (Japan SLC, Shizuoka, Japan) were injected with 3% thioglycollate medium (2 mL/mouse) into the peritoneum. Three days after injection, the mice were sacrificed and injected with 3 mL of 0.05% ethylenediaminetetraacetic acid in phosphate-buffered saline (PBS) into the peritoneum to harvest thioglycollate-elicited peritoneal macrophages. After centrifuging the collected cells at 1200× *g* for 5 min at 4 °C, the cell pellet was washed with PBS and centrifuged again. The cells were then suspended in 10% FBS-RPMI 1640 medium and cultured in 100 mm culture dishes. After incubation at 37 °C for 1 h, the cells were washed with PBS three times to remove unattached cells such as neutrophils. The purity of peritoneal macrophages prepared using this method was estimated to be higher than 85% according to previous reports [41,42,43]. In the subsequent experiments, peritoneal macrophages were detached by pipetting in cold PBS. All animal experiments described herein were carried out in accordance with protocols approved by the Ehime University Animal Care and Use Committee and were performed in accordance with applicable guidelines and regulations for the Care and Use of Laboratory Animals of Ehime University.

### 3.3. Preparation of OSA-PG

OSA-PG was prepared using the method described previously [8] with slight modifications. In brief, PG was extracted from porang tuber harvested by a local farmer at Nglanggeran, Yogyakarta, Indonesia in February 2016, and 10 g of dry PG powder, 1 g of Na_2_CO_3_, and 15 mL of 30% ethanol were mixed under stirring. After mixing homogeneously, 10 mL of 2-octen-1-ylsuccinic anhydride diluted five-fold in absolute ethanol was slowly added with agitation. The mixture was then heated at 90 °C for 20 min, cooled to room temperature, mixed with 40 mL of 30% ethanol for 5 min, and adjusted to pH 6.5–7.0 with 1 M HCl. The mixture was subsequently centrifuged at 3000× *g* for 10 min, and the precipitate was washed with 30% ethanol five times and with absolute ethanol five times. The solid portion was then dried at 40 °C for 24 h and sieved with a 100 mesh nylon sieve. A portion of OSA-PG powder (150 mg) was stirred in 10 mL of distilled water for 20 min and left at 4 °C for 24 h in a rotary shaker. After centrifugation at 20,000× *g* for 20 min, the supernatant was collected and sterilized by autoclave at 121 °C for 20 min. Then, the obtained sample was used as OSA-PG for the experiments described below.

### 3.4. Cytokine Production Assay

The assay was performed using the method described previously [28]. J774.1 cells and peritoneal macrophages were cultured in 96-well culture plates at 1.8 × 10^5^ cells/well and 3.0 × 10^5^ cells/well, respectively, in 10% FBS-RPMI 1640 medium at 37 °C overnight. The cells were then treated with various concentrations of OSA-PG in 10% FBS-RPMI 1640 medium for 6 h at 37 °C, and the concentrations of IL-6 and TNF-α in culture media were measured using ELISA kits. To evaluate an effect of polymyxin B on the activity of OSA-PG, 2.5 mg of OSA-PG or 200 ng of LPS was treated with 10 μg of polymyxin B for 1 h at room temperature. The polymyxin B-treated OSA-PG and polymyxin B-treated LPS were used for the cytokine production assay as described above. Evaluation of the effect of TLR4 inhibition on cytokine production facilitated by OSA-PG was performed using TAK-242, a TLR4 inhibitor. J774.1 cells were cultured in 96-well culture plates at 1.8 × 10^5^ cells/well in 10% FBS-RPMI 1640 medium at 37 °C overnight. The cells were then treated with 0.2 μM TAK-242 in 10% FBS-RPMI 1640 medium for 1 h. After washing with PBS, the cells were treated with 2.5 mg/mL of OSA-PG or 200 ng/mL of LPS (positive control) in 10% FBS-RPMI 1640 medium for 6 h at 37 °C, and the concentrations of TNF-α and IL-6 in culture media were measured by ELISA kits.

### 3.5. Real-Time RT-PCR

J774.1 cells or peritoneal macrophages were cultured in 48-well culture plates at 3.0 × 10^5^ cells/well in 10% FBS-RPMI 1640 medium at 37 °C overnight. The cells were then treated with 2.5 mg/mL of OSA-PG in 10% FBS-RPMI 1640 medium for 6 h at 37 °C. After incubation, total RNA was isolated from the cells using Sepasol-RNA I super G (Nacalai Tesque, Kyoto, Japan) according to the manufacturer’s instructions and used as a template for cDNA synthesis. Real-time RT-PCR was performed as previously described [44]. Briefly, the real-time PCR mixture, with a final volume of 20 μL, consisted of Thunderbird SYBR pPCR Mix (Toyobo, Osaka, Japan), 1 μM of a forward primer, 1 μM of a reverse primer, and 0.1 μg of a cDNA sample. The thermal cycling conditions were: 20 s at 95 °C; and 40 cycles of 3 s at 95 °C and 30 s at 60 °C. The PCR products were performed by StepOnePlus System (Applied Biosystems, Foster City, CA, USA), and the relative gene expression was calculated based on the comparative CT method using StepOne Software v2.1 (Applied Biosystems, Foster City, CA, USA). Expression of the β-actin gene was used as an endogenous control. Specific PCR primer sequences for each gene are shown in Table 1.

### 3.6. Phagocytosis Activity

The assay was performed as described previously [40] with some modifications. J774.1 cells were treated with 2.5 mg/mL of OSA-PG in 10% FBS-RPMI 1640 medium for 6 h. After removing the culture medium, the cells were cultured in RPMI 1640 medium containing Texas Red-labeled zymosan A (*Saccharomyces cerevisiae*) bioparticles (Invitrogen, Carlsbad, CA, USA) for 1 h at 37 °C under the dark condition. The cell pellet was detached by pipetting in PBS and centrifuged at 1000× *g* for 10 min. The pellet was then resuspended in 1 mL of 2% FBS-PBS, and the phagocytosis activity was measured in a flow cytometer (FACSCalibur, BD Biosciences, San Jose, CA, USA).

### 3.7. Immunoblot Analysis

J774.1 cells were cultured in 48-well culture plates at 5.0 × 10^5^ cells/well in 10% FBS-RPMI 1640 medium at 37 °C overnight. After that, the cells were treated with 2.5 mg/mL of OSA-PG or 200 ng/mL of LPS in 10% FBS-RPMI 1640 medium for 30 min at 37 °C. Total cell lysate was prepared as previously described [45]. Cytosolic and nuclear proteins were each isolated using a CelLytic NuCLEAR Extraction kit (Sigma-Aldrich, St. Louis, MO, USA) according to the manufacturer’s instructions. Protein concentration was quantified using a DC Protein Assay kit (Bio-Rad Laboratories, Hercules, CA, USA). Heat-denatured proteins were separated by sodium dodecyl sulfate-polyacrylamide gel electrophoresis and transferred onto a polyvinylidene fluoride membrane (Hybond-P; GE Healthcare, Buckinghamshire, UK). Immunoblotting with various antibodies was performed as previously described [23]. Bands were analyzed using a ChemiDoc XRS Plus apparatus (Bio-Rad Laboratories), and the chemiluminescent intensity was quantified using the Quantity One software (Bio-Rad Laboratories).

### 3.8. Statistical Analysis

Statistical analyses were performed using GraphPad Prism version 7.02 (GraphPad Software, La Jolla, CA, USA). Statistical significance was determined via an unpaired *t*-test or one-way analysis of variance (ANOVA) with Dunnett’s multiple comparison test as indicated. Values with * *p* < 0.05, ** *p* < 0.01, and *** *p* < 0.001 were considered statistically significant.

## 4. Conclusions

The present study demonstrated the immunomodulatory potential of OSA-PG in macrophages. OSA-PG enhanced the phagocytosis activity of macrophages and increased the production of IL-6 and TNF-α by elevating the gene expression levels in both J774.1 cells and peritoneal macrophages. Immunoblot analysis implied that OSA-PG increased cytokine production through the activation of NF-κB and MAPK signaling cascades. In addition, cytokine production induced by OSA-PG was not caused by endotoxin contamination. OSA-PG activated macrophages through not only TLR4 but another receptor. The study indicated that OSA-PG could be used as a functional food with an immunomodulatory effect on macrophages.

## Figures and Tables

**Table 1:** Sequences of primers for real-time RT-PCR.

Primer	Sequence (5′-3′)
TNF-α	5′-CTACTCCCAGGTTCTCTTCAA-3′ (sense)
	5′-GCAGAGAGGAGGTTGACTTTC-3′ (antisense)
IL-6	5′-AAGCCAGAGTCCTTCAGAGAGAT-3′ (sense)
	5′-TTGGATGGTCTTGGTCCTTAGC-3′ (antisense)
β-Actin	5′-CATCCGTAAAGACCTCTATGCCAAC-3′ (sense)
	5′-ATGGAGCCACCGATCCACA-3′ (antisense)

## Supplementary Materials

The following are available online.

## References

[1] Harmayani E., Aprilia V., Marsono Y. (2014). Characterization of glucomannan from *Amorphophallus oncophyllus* and its prebiotic activity in vivo. Carbohydr. Polym..

[2] Yanuriati A., Marseno D.W., Rochmadi, Harmayani E. (2017). Characteristics of glucomannan isolated from fresh tuber of Porang (*Amorphophallus muelleri* Blume). Carbohydr. Polym..

[3] Al-Ghazzewi F.H., Khanna S., Tester R.F., Piggott J. (2007). The potential use of hydrolysed konjac glucomannan as a prebiotic. J. Sci. Food Agric..

[4] Chua M., Baldwin T.C., Hocking T.J., Chan K. (2010). Traditional uses and potential health benefits of *Amorphophallus konjac* K. Koch ex N.E.Br. J. Ethnopharmacol..

[5] Imeson A. (2009). Food Stabilisers, Thickeners and Gelling Agents.

[6] Mikkonen K.S., Tenkanen M., Cooke P., Xu C., Rita H., Willför S., Holmbom B., Hicks K.B., Yadav M.P. (2009). Mannans as stabilizers of oil-in-water beverage emulsions. LWT-Food Sci. Technol..

[7] Zhang Y.Q., Xie B.J., Gan X. (2005). Advance in the applications of konjac glucomannan and its derivatives. Carbohydr. Polym..

[8] Meng F., Zheng L., Wang Y., Liang Y., Zhong G. (2014). Preparation and properties of konjac glucomannan octenyl succinate modified by microwave method. Food Hydrocoll..

[9] Sarkar S., Singhal R.S. (2014). Esterification of guar gum hydrolysate and gum Arabic with *n*-octenyl succinic anhydride and oleic acid and its evaluation as wall material in microencapsulation. Carbohydr. Polym..

[10] Sweedman M.C., Tizzotti M.J., Schäfer C., Gilbert R.G. (2013). Structure and physicochemical properties of octenyl succinic anhydride modified starches: A review. Carbohydr. Polym..

[11] Chen H.M., Fu X., Luo Z.G. (2015). Esterification of sugar beet pectin using octenyl succinic anhydride and its effect as an emulsion stabilizer. Food Hydrocoll..

[12] Cheng J.H., Hu Y.N., Luo Z.G., Chen W., Chen H.M., Peng X.C. (2017). Preparation and properties of octenyl succinate β-cyclodextrin and its application as an emulsion stabilizer. Food Chem..

[13] Tester R.F., Al-Ghazzewi F.H. (2016). Beneficial health characteristics of native and hydrolysed konjac (*Amorphophallus konjac*) glucomannan. J. Sci. Food Agric..

[14] Li B., Xia J., Wang Y., Xie B. (2005). Structure characterization and its antiobesity of ball-milled konjac flour. Eur. Food Res. Technol..

[15] Chen H.L., Sheu W.H.H., Tai T.S., Liaw Y.P., Chen Y.C. (2003). Konjac supplement alleviated hypercholesterolemia and hyperglycemia in type 2 diabetic subjects—A randomized double-blind trial. J. Am. Coll. Nutr..

[16] Al-Ghazzewi F.H., Tester R.F. (2012). Efficacy of cellulase and mannanase hydrolysates of konjac glucomannan to promote the growth of lactic acid bacteria. J. Sci. Food Agric..

[17] Onishi N., Kawamoto S., Suzuki H., Santo H., Aki T., Shigeta S., Hashimoto K., Hide M., Ono K. (2007). Dietary pulverized konjac glucomannan suppresses scratching behavior and skin inflammatory immune responses in NC/Nga mice. Int. Arch. Allergy Immunol..

[18] Suzuki H., Oomizu S., Yanase Y., Onishi N., Uchida K., Mihara S., Ono K., Kameyoshi Y., Hide M. (2010). Hydrolyzed konjac glucomannan suppresses IgE production in mice B cells. Int. Arch. Allergy Immunol..

[19] Hume D.A. (2006). The mononuclear phagocyte system. Curr. Opin. Immunol..

[20] Katsiari C.G., Liossis S.N.C., Sfikakis P.P. (2010). The pathophysiologic role of monocytes and macrophages in systemic lupus erythematosus: A reappraisal. Semin. Arthritis Rheum..

[21] Aderem A., Ulevitch R.J. (2000). Toll-like receptors in the induction of the innate immune response. Nature.

[22] Liao W., Luo Z., Liu D., Ning Z., Yang J., Ren J. (2015). Structure characterization of a novel polysaccharide from *Dictyophora indusiata* and its macrophage immunomodulatory activities. J. Agric. Food Chem..

[23] Wijanarti S., Putra A.B.N., Nishi K., Harmayani E., Sugahara T. (2016). Immunostimulatory activity of snake fruit peel extract on murine macrophage-like J774.1 cells. Cytotechnology.

[24] Che T.M., Johnson R.W., Kelley K.W., Dawson K.A., Moran C.A., Pettigrew J.E. (2012). Effects of mannan oligosaccharide on cytokine secretions by porcine alveolar macrophages and serum cytokine concentrations in nursery pigs. J. Anim. Sci..

[25] Lejeune F.J., Liénard D., Matter M., Rüegg C. (2006). Efficiency of recombinant human TNF in human cancer therapy. Cancer Immun..

[26] Padmore T., Stark C., Turkevich L.A., Champion J.A. (2017). Quantitative analysis of the role of fiber length on phagocytosis and inflammatory response by alveolar macrophages. Biochim. Biophys. Acta.

[27] Sigola L.B., Fuentes A.L., Millis L.M., Vapenik J., Murira A. (2016). Effects of Toll-like receptor ligands on RAW 264.7 macrophage morphology and zymosan phagocytosis. Tissue Cell.

[28] Kumalasari I.D., Nishi K., Putra A.B.N., Sugahara T. (2014). Activation of macrophages stimulated by the bengkoang fiber extract through toll-like receptor 4. Food Funct..

[29] Putra A.B.N., Nishi K., Shiraishi R., Doi M., Sugahara T. (2014). Jellyfish collagen stimulates production of TNF-α and IL-6 by J774.1 cells through activation of NF-κB and JNK via TLR4 signaling pathway. Mol. Immunol..

[30] Lee S.G., Jung J.Y., Shin J.S., Shin K.S., Cho C.W., Rhee Y.K., Hong H.D., Lee K.T. (2015). Immunostimulatory polysaccharide isolated from the leaves of *Diospyros kaki* Thumb modulate macrophage via TLR2. Int. J. Biol. Macromol..

[31] Xu X., Wu X., Wang Q., Cai N., Zhang H., Jiang Z., Wan M., Oda T. (2014). Immunomodulatory effects of alginate oligosaccharides on murine macrophage RAW264.7 cells and their structure-activity relationships. J. Agric. Food Chem..

[32] Davis R.J. (2000). Signal transduction by the JNK group of MAP kinases. Cell.

[33] Xia Y., Makris C., Su B., Li E., Yang J., Nemerow G.R., Karin M. (2000). MEK kinase 1 is critically required for c-Jun N-terminal kinase activation by proinflammatory stimuli and growth factor-induced cell migration. Proc. Natl. Acad. Sci. USA.

[34] Esteban E., Ferrer R., Alsina L., Artigas A. (2013). Immunomodulation in sepsis: The role of endotoxin removal by polymyxin B-immobilized cartridge. Mediators Inflamm..

[35] Lei W., Browning J.D., Eichen P.A., Lu C.H., Mossine V.V., Rottinghaus G.E., Folk W.R., Sun G.Y., Lubahn D.B., Fritsche K.L. (2015). Immuno-stimulatory activity of a polysaccharide-enriched fraction of *Sutherlandia frutescens* occurs by the toll-like receptor-4 signaling pathway. J. Ethnopharmacol..

[36] Gordon S. (2002). Pattern recognition receptors: Doubling up for the innate immune response. Cell.

[37] Xu X., Yan H., Zhang X. (2012). Structure and immuno-stimulating activities of a new heteropolysaccharide from *Lentinula edodes*. J. Agric. Food Chem..

[38] Ma P., Liu H.T., Wei P., Xu Q.S., Bai X.F., Du Y.G., Yu C. (2011). Chitosan oligosaccharides inhibit LPS-induced over-expression of IL-6 and TNF-α in RAW264.7 macrophage cells through blockade of mitogen-activated protein kinase (MAPK) and PI3K/Akt signaling pathways. Carbohydr. Polym..

[39] Cai H.L., Huang X.J., Nie S.P., Xie M.Y., Phillips G.O., Cui S.W. (2015). Study on *Dendrobium officinale* O-acetyl-glucomannan (Dendronan^®^): Part III-immunomodulatory activity in vitro. Bioact. Carbohydr. Diet. Fibre.

[40] Putra A.B.N., Morishige H., Nishimoto S., Nishi K., Shiraishi R., Doi M., Sugahara T. (2012). Effect of collagens from jellyfish and bovine Achilles tendon on the activity of J774.1 and mouse peritoneal macrophage cells. J. Funct. Foods.

[41] Ding A.H., Nathan C.F., Stuehr D.J. (1988). Release of reactive nitrogen intermediates and reactive oxygen intermediates from mouse peritoneal macrophages. Comparison of activating cytokines and evidence for independent production. J. Immunol..

[42] Vodovotz Y., Bogdan C., Paik J., Xie Q.W., Nathan C. (1993). Mechanisms of suppression of macrophage nitric oxide release by transforming growth factor β. J. Exp. Med..

[43] Schindler H., Lutz M.B., Röllinghoff M., Bogdan C. (2001). The production of IFN-γ by IL-12/IL-18-activated macrophages requires STAT4 signaling and is inhibited by IL-4. J. Immunol..

[44] Nishi K., Kondo A., Okamoto T., Nakano H., Daifuku M., Nishimoto S., Ochi K., Takaoka T., Sugahara T. (2011). Immunostimulatory in vitro and in vivo effects of a water-soluble extract from kale. Biosci. Biotechnol. Biochem..

[45] Kanda K., Nishi K., Kadota A., Nishimoto S., Liu M.C., Sugahara T. (2012). Nobiletin suppresses adipocyte differentiation of 3T3-L1 cells by an insulin and IBMX mixture induction. Biochim. Biophys. Acta.

