# Supplementary material for: Immunomodulatory Activity of Octenyl Succinic Anhydride Modified Porang (Amorphophallus oncophyllus) Glucomannan on Mouse Macrophage-Like J774.1 Cells and Mouse Primary Peritoneal Macrophages
Source: Molecules. 2017 Jul 15;22(7):1187. doi: 10.3390/molecules22071187 (PMC6152250; doi:10.3390/molecules22071187)
Supplement: Supplementary file 1 [file molecules-22-01187-s001.pdf]

Supplementary material

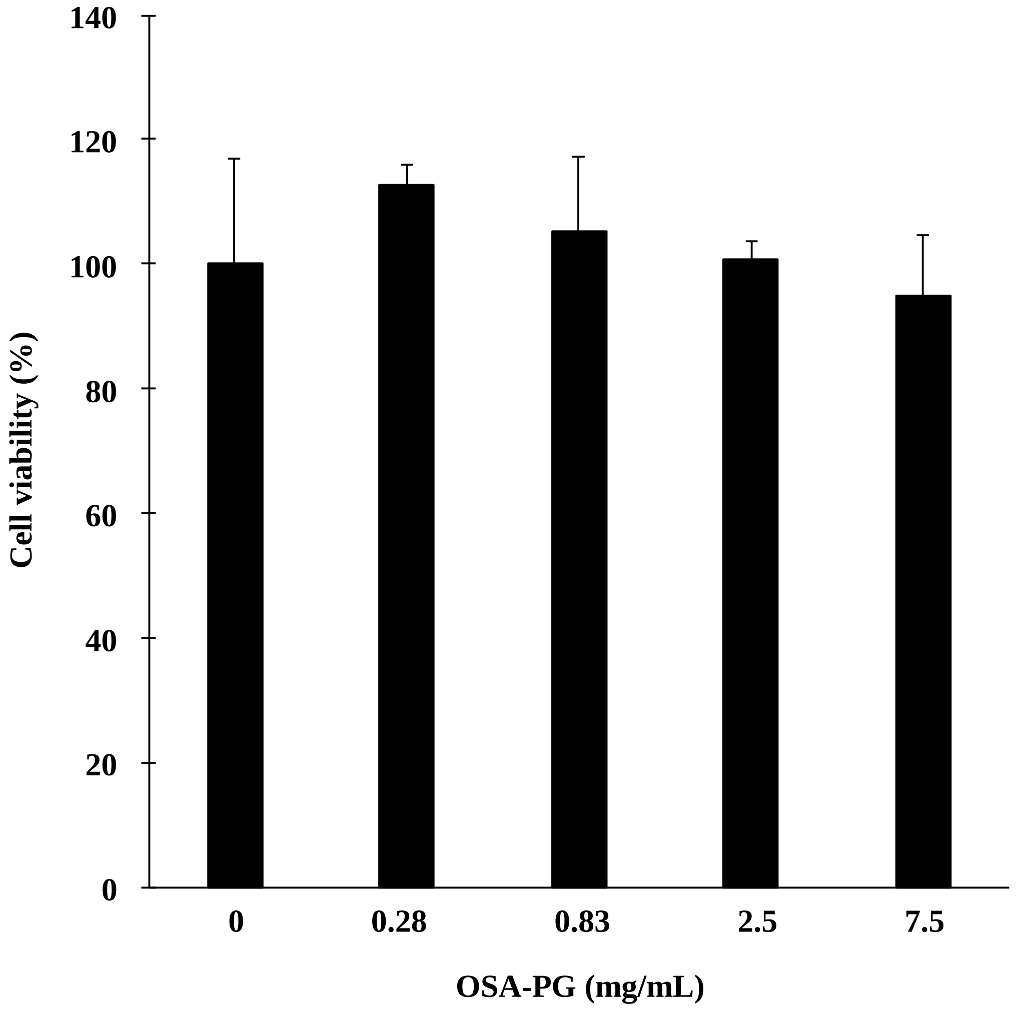

Effect of octenyl succinic anhydride-modified porang glucomannan (OSA-PG) on the viability of J774.1 cells. Viability of J774.1 cells treated with various concentrations of OSA-PG was evaluated by the WST-8 assay. J774.1 cells were cultured in 96-well culture plates at 3.0×10^5 cells/mL in 10% FBS-RPMI 1640 medium at 37 ºC overnight. The cells were then treated with various concentrations of OSA-PG in 1640 medium containing the 10% WST-8 solution for 20 min at 37 ºC. The absorbance at 450 nm was then measured in a microplate reader. Results are shown as mean ± standard deviation in triplicate.
